# Re-hospitalisation predicts poor prognosis after acute exacerbation of interstitial lung disease

**DOI:** 10.1186/s12890-023-02534-0

**Published:** 2023-07-01

**Authors:** Johanna Salonen, Sanna Jansa, Hannu Vähänikkilä, Riitta Kaarteenaho

**Affiliations:** 1 Research Unit of Biomedicine and Internal Medicine, University of Oulu, Oulun Yliopisto, P.O. Box 8000, 90014 Oulu, Finland; 2 Center of Internal and Respiratory Medicine and Medical Research Center (MRC) Oulu, Oulu University Hospital, Oulu, Finland; 3 Northern Finland Birth Cohorts, Arctic Biobank, Infrastructure for Population Studies, Faculty of Medicine, University of Oulu, Oulu, Finland

**Keywords:** Idiopathic pulmonary fibrosis, Interstitial lung disease, Acute exacerbation, Survival

## Abstract

**Background:**

Several markers have been identified to increase the risk for acute exacerbation of interstitial lung disease (AE-ILD) or mortality related to AE-ILD. However, less is known about the risk predictors of ILD patients who have survived AE. The aim of the study was to characterise AE-ILD survivors and investigate prognostic factors in this subpopulation.

**Methods:**

All AE-ILD patients (*n* = 95) who had been discharged alive from two hospitals located in Northern Finland were selected from a population of 128 AE-ILD patients. Clinical data related to the hospital treatment and six-month follow-up visit were collected retrospectively from medical records.

**Results:**

Fifty-three patients with idiopathic pulmonary fibrosis (IPF) and 42 patients with other ILD were identified. Two thirds of the patients had been treated without invasive or non-invasive ventilation support. The clinical features of six-month survivors (*n* = 65) and non-survivors (*n* = 30) did not differ in terms of medical treatment or oxygen requirements. Of the patients, 82.5% used corticosteroids at the six-month follow-up visit. Fifty-two patients experienced at least one non-elective respiratory re-hospitalisation before the six-month follow-up visit. In a univariate model, IPF diagnosis, high age and a non-elective respiratory re-hospitalisation increased the risk of death, although re-hospitalisation was the only independent risk factor in a multivariate model. In six-month survivors, there was no statistically significant decrease in pulmonary function test results (PFT) examined at the follow-up visit compared with earlier PFT examined near the time of AE-ILD.

**Conclusions:**

The AE-ILD survivors were a heterogeneous group of patients both clinically and in terms of their outcome. A non-elective respiratory re-hospitalisation was identified as a marker of poor prognosis among AE-ILD survivors.

## Background

Interstitial lung diseases are a group of more than 200 disorders of the lung parenchyma which heterogenous pathological, radiological and clinical features [1–4]. Acute exacerbation of interstitial lung disease (AE-ILD) is associated with poor survival time in both idiopathic pulmonary fibrosis (IPF) and in other types of interstitial lung disease (ILD) [5–13].

The antifibrotic drugs pirfenidone and nintedanib slow down the progression of IPF and other types of fibrotic ILDs with acceptable safety profiles, which has been proved also in real-life study settings [14–16]. Both antifibrotic drugs seem to prevent AE-ILDs and reduce the number of acute respiratory hospitalisations in ILD patients [17, 18]. These benefits might be related to the immune-modulative effects of the antifibrotic drugs on the processes present at the development of AE-ILD [19–21]. It is noteworthy that in a significant proportion of patients, AE-ILD can be the first manifestation of ILD when the patients have not been able to benefit from the preventive effects of antifibrotic drugs [5, 10]. The conventional treatment of AE-ILD has been glucocorticoids and other immunosuppressants, although there is a lack of randomized, controlled studies on the efficacy of these treatments [5, 6]. There has been even concern about the potential harmfulness of the glucocorticoid treatment in AE-ILD [22].

Several parameters have been identified to predict the occurrence of AE-ILD or the mortality of AE-ILD patients. These include, for example, low pulmonary function test results (PFT) or enhanced rate of decline in PFT, high age, male gender, high body mass index (BMI) or usual interstitial pneumonia (UIP) pattern in high-resolution computed tomography (HRCT) [23–26]. The factors indicating a more severe respiratory failure, such as the need for invasive or non-invasive ventilation support or low rate of arterial oxygen partial pressure to fractional inspired oxygen (P/F ratio), have been reported to increase the mortality of AE-ILD patients [10, 25–30]. In recent studies, 3-month mortality in AE-ILD has been about 40 − 50%, independent of the ILD type [24–26, 28, 31].

As previously described, most investigations reporting the clinical features and prognostic factors of AE-ILD patients have not further described the characteristics and outcome of AE-ILD-survivors, their medical treatment after hospital discharge and follow-up data on PFT after AE-ILD [7–11, 13, 23–31]. Our study aimed to characterise the patients who had been treated in Oulu University Hospital (OUH) or Oulaskangas Hospital (OH) in Northern Finland during 2008 − 2017 and survived AE-ILD. We collected the data related to the hospital treatment caused by AE-ILD and the follow-up visit about 6 months after discharge. Age, gender, PFT, pharmacological therapy, requirement of ventilation support and/or supplementary oxygen, non-elective respiratory re-hospitalisations and survival data were collected. The characteristics of patients with AE-ILD with less than 6 months’ survival time were compared with those with longer survival time.

## Methods

### Patient and data collection

**Fig. 1:**
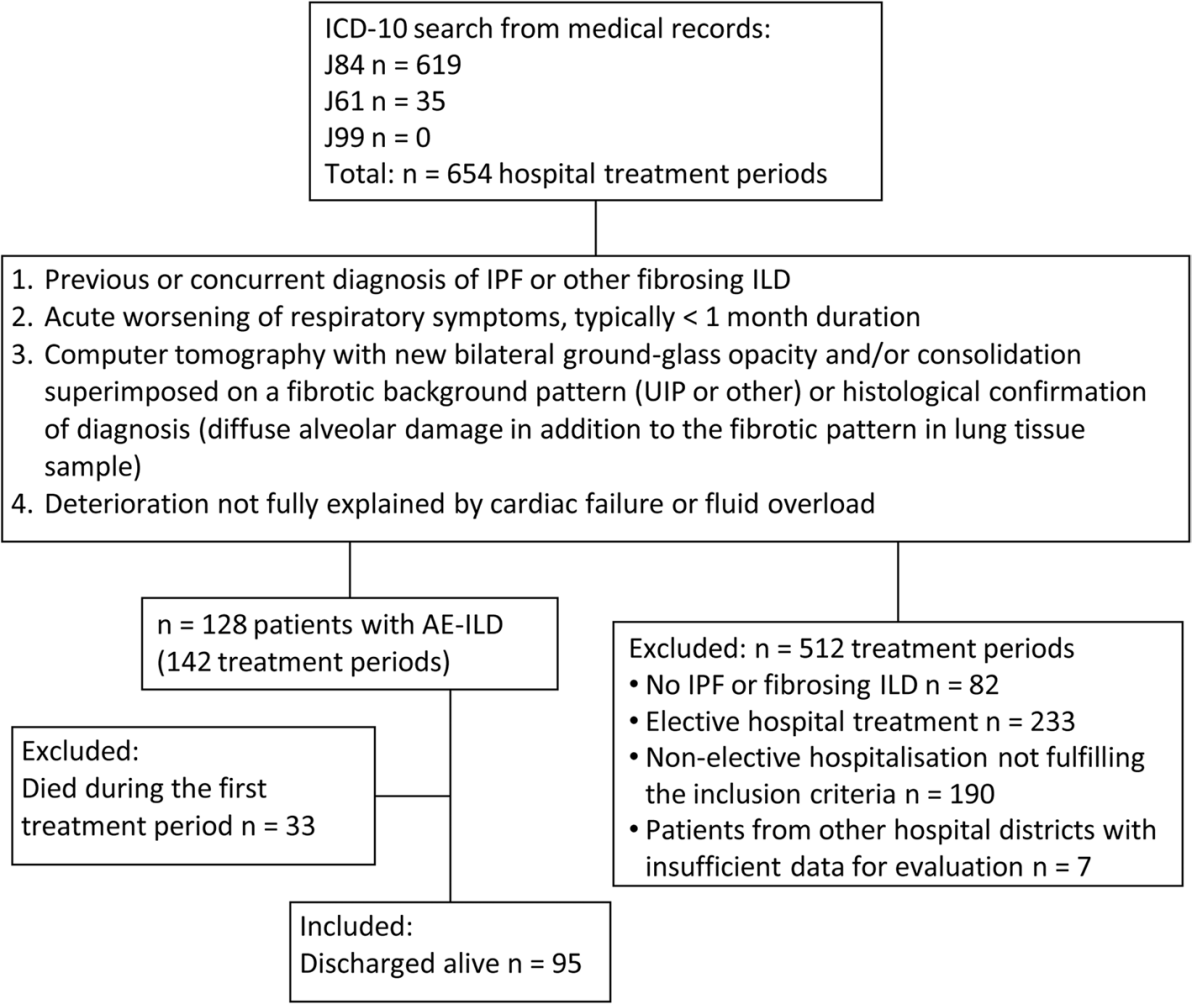
Flow chart of the study. Abbreviations: AE-ILD, acute exacerbation of interstitial lung disease; ICD-10, International Classification of Diseases, Tenth Revision; ILD, interstitial lung disease; IPF, idiopathic pulmonary fibrosis

The flow chart of the study is presented in Fig. 1. All patients of this study were picked up from our previous study comprising 128 AE-ILD patients treated in OUH or OH in 2008 − 2017 [10]. Ninety-five patients who had been discharged alive after their first episode of AE-ILD were included and 33 AE-ILD patients who had died during their hospital treatment period were excluded. The patients were originally searched with International Classification of Diseases, Tenth Revision (ICD-10) diagnosis codes J84.1 J84.8 and J84.9, aiming at finding patients with IPF (mostly coded with J84.1) and non-IPF ILDs (codes J84.1, J84.8 and J84.9) (1) [32]. An additional search was performed with codes J61, J99, J99.0* and J99*M05.1 to find the patients with asbestosis (J61) and connective-tissue disease-associated ILDs (J99.0*), especially rheumatoid arthritis-associated ILDs (RA-ILD) (J99*M05.1) (Table 1) [32]. Concerning the additional search, only J61 produced matches. The type of ILD was re-evaluated according to the international criteria as described in detail in our previous study [10, 33, 34]. The definition of AE-IPF by Collard et al.(2016) was utilised and applied to all patients, including those with non-IPF ILDs [5]. The definition of AE-ILD included 1) acute respiratory symptoms of approximately less than one month’s duration, 2) new bilateral consolidation/ground glass opacities in chest HRCT in addition to chronic fibrotic changes (UIP or other type of fibrotic changes), and 3) no explanatory alternative diagnosis. The clinical information was collected retrospectively from medical records. The dates of death were collected from death certificates housed in the national registry of Statistics Finland. The survival time was calculated from hospitalisation date to date of death, lung transplantation, or last follow-up date (31^st^ August 2019).

**Table 1:** ICD-10 diagnosis codes utilised in the search of AE-ILD patients included in the study [32]

ICD-10 code	Diagnosis
J84.1	Other interstitial pulmonary diseases with fibrosis
J84.8	Other specified interstitial pulmonary diseases
J84.9	Interstitial pulmonary disease, unspecified
J61	Pneumoconiosis due to asbestos and other mineral fibres
J99	Respiratory disorders in diseases classified elsewhere
J99.0	Rheumatoid lung disease
J99*M05.1	Rheumatoid lung disease

*Abbreviations*: *ICD-10* International Classification of Diseases, Tenth Revision

A large proportion of data was already collected during the implementation of our earlier study, which is described in detail elsewhere [10]. Specifically for this study, we collected some additional information concerning the hospital treatment period related to AE-ILD and the follow-up visit that took place approximately 6 months after the first episode of AE-ILD from electronic medical records. The collected data included the form of ventilation support and supplementary oxygen requirement during the hospital treatment period, BMI, need for supplementary oxygen or home-oxygen therapy at hospital discharge, discharge disposition, non-elective respiratory re-hospitalisations and their causes, and pharmacotherapy of ILD. Readmissions after AE-ILD within three months were not regarded as new, separate AE-ILDs, if the clinical presentation of ILD had not been stabilised in that time frame and a new episode meeting criteria of AE could not be confirmed. There were three patients for whom the follow-up data was collected from a non-elective hospital treatment period following the episode of AE-ILD. There were also two patients who had been treated in another hospital after discharge and on whom we were not able to collect detailed follow-up data. However, these patients were included in the analysis because the data concerning the hospital treatment period and survival time were available.

### Statistical analysis

The statistical analysis was performed with SPSS (IBM Corp. Released 2020. IBM SPSS Statistics for Windows, Version 27.0. Armonk, NY: IBM Corp) and OriginPro was utilised for graphs (Version 2022. OriginLab Corporation, Northampton, MA, USA). The categorical clinical parameters were reported as the frequencies and percentages of patients. The chi-square test or Fisher’s exact test were utilised in the comparison of categorical values. For normally distributed, continuous values, mean and standard deviation were reported, and independent sample or paired sample T-test were used for comparison of these values. Not-normally distributed values were reported as medians and minimum − maximum values, and the groups were compared with each other by Mann–Whitney U-test. Kaplan–Meier curve was performed to estimate median survival time of AE-ILD patients and log rank test was utilised to compare the survival time of different groups. Risk of mortality was evaluated by using Cox regression model. Complete case analysis was used to deal with variables with missing data.

## Results

### Characteristics of AE-ILD patients who had survived the hospital treatment period

There were 95 AE-ILD patient who were discharged alive from hospital (Table 2). Table 2 presents these patients according to their survival status at six months after the hospitalisation date. More than half of the patients had IPF (53/95), and 20 of 53 IPF patients died less than 6 months after the hospitalisation. In contrast, most patients with either rheumatoid arthritis-associated ILD (RA-ILD), non-specific interstitial pneumonia (NSIP) or other ILD had longer than 6 months’ survival time. There were no statistically significant differences in oxygen requirements while in hospital, treatment disposition at discharge, or medical treatment between 6-month survivors and non-survivors. There were more cases without earlier ILD diagnosis among 6-month survivors compared with non-survivors. Furthermore, non-elective respiratory re-hospitalisations were more common among patients with less than 6 months’ survival time compared with those with a longer survival.

**Table 2:** The patients discharged from hospital according to survival status six months after AE-ILD

Parameter at discharge date	Total *n* = 95	Survived six months *n* = 65	Deceased in six months *n* = 30	*P*-value
Male gender	61 (64.2)	41 (63.1)	20 (66.7)	0.734
Age (years)	72.8 (9.1)	72.5 (9.7)	73.4 (7.7)	0.653
ILD diagnosed at the time of hospitalisation	29 (30.5)	26 (39.4)	3 (10.3)	0.005
ILD type				
IPF	53 (55.8)	33 (50.8)	20 (66.7)	0.147
RA-ILD	15 (15.8)	13 (20.0)	2 (6.7)	0.133
Asbestosis	9 (9.5)	5 (7.7)	4 (13.3)	0.457
NSIP	8 (8.4)	6 (9.2)	2 (6.7)	> 0.999
Other	10 (10.5)	8 (12.3)	2 (6.7)	0.496
Oxygen requirement during hospital treatment				
None	3 (3.2)	2 (3.1)	1 (3.3)	0.541
Nasal cannula	62 (65.3)	44 (67.7)	18 (60.0)	
HFNO	4 (4.2)	2 (3.1)	2 (6.7)	
CPAP	5 (5.3)	3 (4.6)	2 (6.7)	
Non-invasive ventilation	12 (12.6)	6 (9.2)	6 (20.0)	
Intubation	4 (4.2)	4 (6.2)	0	
Discharge disposition				
Home	53 (55.8)	39 (60.0)	14 (46.7)	0.224
Hospital ward in primary care	42 (44.2)	26 (40.0)	16 (53.3)	0.224
Medical treatment at discharge				
Corticosteroid	91 (95.8)	61 (93.8)	30 (100.0)	0.304
Corticosteroid dose (mg)^a^	31.3 (13.5)	31.7 (1.7)	30.5 (14.2)	0.698
Antifibrotic drug at discharge (total)	7 (7.4)	5 (7.7)	2 (6.7)	> 0.999
Pirfenidone	5 (5.3)	3 (4.6)	2 (6.7)	
Nintedanib	2 (2)	2 (2.1)	0	
Other immunosuppressant at discharge (total)	7 (7.4)	6 (9.2)	1 (3.3)	0.426
Azathioprine	3 (3.2)	2 (3.1)	1 (3.3)	
Mycophenolate	0	0	0	
Cyclophosphamide	4 (4.2)	4 (6.2)	0	
N-acetylcysteine	6 (6.3)	3 (4.6)	3 (10.0)	0.376
Supplementary oxygen needed	57 (60.0)	36 (55.4)	21 (70.0)	0.176
Home oxygen therapy initiated	38 (40.0)	26 (40.0)	12 (40.0)	> 0.999
Supplementary oxygen rate (l per min)	3 (1 − 10)	2 (1 − 10)	3 (1 − 7)	0.168
Non-elective respiratory re-hospitalisation	52 (55.9)	28 (44.4)	24 (80.0)	0.001
Time from hospital discharge to re-hospitalisation (days)	38 (2 − 260)	71.0 (5 − 260)	22.5 (2 − 102)	0.001
Follow-up time (months)^b^	18.8 (0.3 − 159)	28.9 (6.1 − 159)	2.0 (0.3 − 5.9)	< 0.001
Deceased or transplanted during the follow-up	77 (81.1)	47 (72.3)	30 (100)	0.001

Data are expressed as number of cases (%), mean (standard deviation), or median (minimum − maximum)

*Abbreviations*: *AE-ILD* Acute exacerbation of interstitial lung disease, *CPAP* Continuous positive airway pressure, *HFNO* High-flow nasal oxygen, *ILD* Interstitial lung disease, *NSIP* Non-specific interstitial pneumonia, *RA-ILD* Rheumatoid arthritis-associated interstitial lung disease

^a^Patients with no corticosteroid treatment at discharge excluded

^b^Time from hospitalisation date to death, lung transplantation or last follow-up date

### Clinical features of six-month survivors

Sixty-five AE-ILD patients, 33 of whom had IPF and 32 other ILD, had a survival time of at least 6 months after their first episode of AE-ILD (Table 3). The IPF and non-IPF subgroups did not differ significantly by clinical features, although IPF patients had had higher body mass index during their hospital treatment compared with other ILD patients. However, this difference could no longer be observed at the follow-up visit. The majority of patients had not used mechanical ventilation support (invasive or non-invasive) or high-flow nasal oxygen treatment during their hospital treatment. Patients with IPF tended to have more often re-hospitalisations (18/33) compared with non-IPF patients (10/32), although the difference was not statistically significant. PFT did not differ between IPF and other ILD patients at the follow-up visit (Table 3). However, in the subgroup of patients with a new ILD diagnosis at the time of AE-ILD, PFT were higher compared with other survivors at six-month control visit: mean FVC% predicted was 71.0 with standard deviation (SD) of 17 compared with 60.0 with SD of 14, respectively (*p* = 0.041).

**Table 3:** Characteristics of the patients with at least 6 months’ survival time after AE-ILD

Parameter	Total (*n* = 65)	IPF (*n* = 33)	Other ILD (*n* = 32)	*P*-value
Male gender	41 (63.1)	22 (66.7)	19 (59.4)	0.612
Age at hospitalisation (years)	72.5 (9.7)	73.8 (8.7)	71.2 (10.6)	0.289
Age at follow-up visit	73.2 (9.6)	74.2 (8.8)	72.2 (10.4)	0.409
ILD diagnosed first time during AE-ILD	26 (30.0)	11 (33.3)	15 (46.9)	0.265
Time from hospital discharge to follow-up visit (months)^a^	5.8 (2.8 − 12.2)	5.9 (3.6 − 11.1)	5.6 (2.8 − 12.2)	0.360
Body mass index measured during hospital treatment^b^	28.1 (4.1)	30.1 (5.7)	26.3 (3.5)	0.010
Body mass index at follow-up^c^	29.1 (5.5)	30.2 (6.1)	28.1 (4.7)	0.228
Oxygen requirement during hospital treatment				
None	2 (3.1)	1 (3.0)	1 (3.1)	0.542
Nasal cannula	44 (67.7)	24 (72.7)	20 (62.5)	
Oxygen mask	2 (3.1)	1 (3.0)	1 (3.1)	
HFNO	4 (6.2)	2 (6.1)	2 (6.3)	
CPAP	3 (4.6)	0	3 (9.4)	
Non-invasive ventilation	6 (9.2)	2 (6.1)	4 (12.5)	
Intubation	4 (6.2)	3 (9.1)	1 (3.1)	
Intubation or non-invasive mechanical ventilation support during hospital treatment^d^	13 (20.0)	5 (15.2)	8 (25.0)	0.321
≥ 1 non-elective respiratory re-hospitalisation before the follow-up visit^a^	28 (44.4)	18 (56.3)	10 (32.3)	0.055
Non-elective respiratory re-hospitalisations^a^	0 (0 − 4)	0 (0 − 3)	0 (0 − 3)	0.246
Time from hospital discharge to respiratory re-hospitalisation (days)^a^	71 (5 − 260)	72 (5 − 211)	47 (5 − 260)	0.649
Home oxygen therapy at follow-up^a^	35 (55.6)	21 (65.6)	14 (45.2)	0.102
Supplementary oxygen rate (l per min)	2 (1 − 8)	2 (1 − 6)	3 (1 − 8)	0.200
Pulmonary function test results at follow-up				
VC% of predicted^e^	61.9 (18.1)	62.2 (15.4)	61.7 (20.7)	0.938
FVC% of predicted^f^	65.0 (17.7)	66.7 (16.3)	63.5 (19.2)	0.551
FEV1% of predicted^f^	68.6 (17.5)	70.7 (16.3)	66.6 (18.8)	0.438
FEV1/FVC^g^	83.5 (5.9)	83.8 (5.9)	83.2 (6.0)	0.708
DLCO% of predicted^h^	42.5 (15.3)	39.5 (14.6)	45.2 (15.8)	0.259

Data are expressed as number of cases (%), mean (standard deviation), or median (minimum − maximum)

*Abbreviations*: *AE-ILD* Acute exacerbation of interstitial lung disease, *CPAP* Continuous positive airway pressure, *DLCO* Diffusion capacity for carbon monoxide, *FEV1* Forced expiratory volume in the first second; FVC, forced vital capacity; HFNO, high-flow nasal oxygen; ILD, interstitial lung disease; IPF, idiopathic pulmonary fibrosis; NIV, non-invasive ventilation; VC, vital capacity

^a^The detailed follow-up data of two survivors were missing, because 1 IPF and 1 other ILD patient were treated in a different hospital. However, survival data of these patients was available

^b^Data of 10 IPF and 7 other ILD patients were missing

^c^Data of 14 IPF and 11 other ILD patients were missing

^d^Either CPAP, non-invasive ventilation support or invasive mechanical ventilation

^e^Data of 14 IPF and 12 other ILD patients were missing

^f^Data of 12 IPF and 9 other ILD patients were missing

^g^Data of 11 IPF and 9 other ILD patients were missing

^h^Data of 15 IPF and 12 other ILD patients were missing

### Medical treatment, home oxygen therapy and PFT of six-month survivors

Medical treatment of AE-ILD survivors is presented in Table 4. Almost all patients had been treated with corticosteroids after the hospital discharge (61/63). Of IPF patients, 72%, and of patients with other ILD, 94% still continued corticosteroid treatment after the follow-up visit.

**Table 4:** Medical treatment of AE-ILD in patients with a survival time of at least six months

Parameter	Total *n* = 63	IPF *n* = 32	Other ILD *n* = 31
Corticosteroid therapy at discharge	59 (93.7)	31 (96.9)	28 (90.3)
Corticosteroid initiated after discharge	2 (3.2)	0	2 (6.5)
Corticosteroid therapy finished before the follow-up visit	9 (14.3)	8 (25.0)	1 (3.2)
Duration of therapy (months)	2.0 (0.5 − 5.5)	2.0 (0.5 − 5.5)	1.0
Corticosteroid therapy at follow-up visit^a^	52 (82.5)	23 (71.9)	29 (93.5)
Corticosteroid dosage prescribed at follow-up (mg)	10.0 (2.5 − 50)	10.0 (2.5 − 50.0)	10.0 (2.5 − 30.0)
Antifibrotic treatment at follow-up visit^b^			
Pirfenidone	4 (6.3)	4 (12.5)	0
Nintedanib	2 (3.2)	2 (6.3)	0
Other immunosuppressant at follow-up visit			
Azathioprine	4 (6.3)	2 (6.3)	2 (6.5)
Cyclophosphamide	1 (1.6)	0	1 (3.2)
Mycophenolate	0	0	0
N-acetylcysteine at follow-up visit	2 (3.2)	2 (6.3)	0
No medical treatment of ILD at follow-up visit	9 (14.3)	7 (21.9)	2 (6.5)

Data are expressed as number of cases (%) or median (minimum − maximum)

*Abbreviations*: *AE-ILD* Acute exacerbation of interstitial lung disease, *ILD* Interstitial lung disease, *IPF* Idiopathic pulmonary fibrosis

^a^All patients who used corticosteroid therapy at follow-up visit continued this therapy afterwards

^b^Two patients with pirfenidone and one patient with nintedanib had used this medication already before AE-IPF. Three patients had initiated antifibrotic drug use during the follow-up period

Home oxygen therapy had been initiated for 6 IPF and 3 other ILD patients who had been discharged without supplementary oxygen. In contrast, 3 IPF and 7 non-IPF patients had been able to finish the use of supplementary oxygen before their 6-month follow-up visit. Follow-up data of PFT were available from about half of the 6-month survivors (Table 5). No significant decline could be observed in PFT results after AE-ILD.

**Table 5:** Patients with AE-ILD with 6-month follow-up data of pulmonary function test results

Parameter	Mean (SD)	*P*-value (paired sample T-test)
VC% of pred. (*n* = 32^a^)		0.158
AE-ILD	66.2 (15.6)	
follow-up	63.3 (17.4)	
FVC% of pred. (*n* = 38^b^)		0.780
AE-ILD	67.2 (16.9)	
follow-up	66.6 (16.7)	
FEV1% of pred. (*n* = 38^b^)		0.538
AE-ILD	71.2 (16.5)	
follow-up	70.0 (16.9)	
FEV1/FVC (*n* = 39^c^)		0.069
AE-ILD	84.9 (6.0)	
follow-up	83.2 (5.8)	
DLCO% of pred. (*n* = 35^d^)		0.912
AE-ILD	41.9 (15.9)	
follow-up	42.1 (15.4)	

^a^19 IPF, 13 other ILD

^b^21 IPF, 17 other ILD

^c^22 IPF, 17 other ILD

^d^18 IPF, 17 other ILD

*Abbreviations*: *AE-ILD* Acute exacerbation of interstitial lung disease, *DLCO* Diffusion capacity for carbon monoxide, *FEV1* Forced expiratory volume in the first second, *FVC* Forced vital capacity, *ILD* Interstitial lung disease, *IPF* Idiopathic pulmonary fibrosis, *SD* Standard deviation, *VC* Vital capacity

### Causes of re-hospitalisations and death

The most typical cause of re-hospitalisation was clinical and radiologic progression of AE-ILD, which usually occurred during the first three months after the hospital discharge (Table 6). Two patients recovered from the first episode of AE-ILD and developed a new episode of AE-ILD before the follow-up visit. Lower respiratory tract infection caused about a quarter of readmissions.

**Table 6:** Causes of respiratory re-hospitalisations

Cause of respiratory re-hospitalisation	Total (*n* = 52)
New episode of AE-ILD^a^	2 (3.8)
Clinical-radiologic progression of earlier AE-ILD	27 (51.9)
Lower respiratory tract infection	14 (26.9)
Heart failure	4 (7.7)
Progression of chronic ILD	3 (5.8)
Pneumothorax	1 (1.9)
Multiple causes^b^	1 (1.9)

^a^Readmissions within 3 months were not regarded as new, separate AE-ILDs, if the clinical presentation of ILD had not been stabilised in that time frame and a new episode meeting criteria of AE could not be confirmed

^b^Progression of earlier AE-ILD, gastrointestinal tract hemorrhage and staphylococcus aureus septicaemia

Of the 30 deaths during the 6-month follow-up, ILD was the underlying cause of death in 26 cases. The other causes of deaths were stroke, lung cancer, Hodgkin’s lymphoma and drowning. The immediate causes of deaths were respiratory related almost in all cases, being ILD (13/30), pneumonia (11/30), AE-ILD or acute respiratory distress syndrome (4/30), lung cancer (1/30) or other infection (1/30).

### Respiratory re-hospitalisation was an independent risk factor for death

**Fig. 2:**
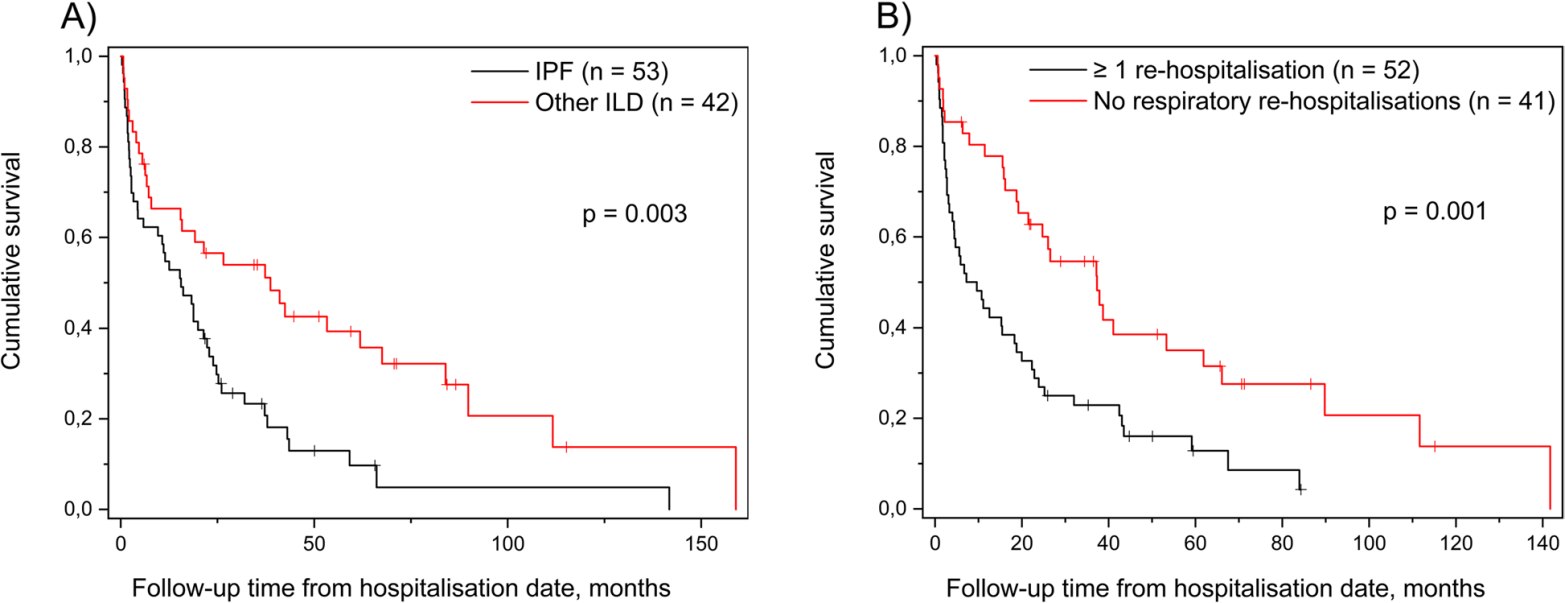
**A** AE-IPF survivors had shorter survival compared with patients who survived AE of other ILD. **B** Non-elective respiratory re-hospitalisation was associated with increased mortality of AE-ILD survivors. Abbreviations: AE-ILD, acute exacerbation of interstitial lung disease; ILD, interstitial lung disease; IPF, idiopathic pulmonary fibrosis

The median survival time of all AE-ILD patients who were discharged alive was 19.2 months with 95% Confidence Interval (CI) of 12.9 − 25.5 months. IPF patients had shorter survival compared with other ILD patients, median survival being 15.6 months (95% CI 8.7 − 22.4) and 38.7 months (95% CI 15.3 − 62.1), respectively (Fig. 2A). Survival time of AE-ILD patients with at least one non-elective respiratory re-hospitalisation before the six-month follow-up visit was significantly shorter compared with the patients with no re-hospitalisations, namely 7.2 months (95% CI 0.7 − 13.7 months) compared with 37.3 months (95% CI 21.7 − 52.9 months), respectively (Fig. 2B).

In Cox Regression analysis, respiratory re-hospitalisation was a poor prognostic factor in both univariate and multivariate model (Table 7). IPF and age were also poor prognostic factors in univariate model, but not in multivariate model.

**Table 7:** Risk for mortality in survivors of acute exacerbation of interstitial lung disease

Parameter	Univariate analysis		Multivariate analysis^a^	
	HR (95% CI)	*p*-value	HR (95% CI)	*p*-value
IPF	2.06 (1.27 − 3.32)	0.003	1.64 (0.99 − 2.72)	0.053
Age	1.04 (1.00 − 1.08)	0.026	1.02 (0.99 − 1.05)	0.246
Male gender	1.21 (0.75 − 1.95)	0.446	1.10 (0.66 − 1.82)	0.721
Non-elective respiratory re-hospitalisation(s) before follow-up visit	2.22 (1.36 − 3.61)	0.001	2.08 (1.25 − 3.45)	0.005
Glucocorticoid dose at least 30 mg per day at hospital discharge	0.81 (0.51 − 1.27)	0.357	0.68 (0.42 − 1.10)	0.112
Discharge disposition home	0.85 (0.54 − 1.34)	0.490	1.11 (0.65 − 1.89)	0.697
Supplementary oxygen needed at discharge	1.62 (1.00 − 2.62)	0.050	1.42 (0.86 − 2.32)	0.171
DLCO% predicted	0.996 (0.98 − 1.01)	0.648	0.994 (0.98 − 1.01)	0.524
FVC% predicted	0.997 (0.981 − 1.014)	0.739	0.802 (0.53 − 1.21)	0.292

^a^Multivariate analysis included IPF, age, non-elective respiratory re-hospitalisation in 12 months, supplementary oxygen needed at discharge. Abbreviations: *CI* Confidence interval, *DLCO* Diffusion capacity for carbon monoxide, *FVC* Forced vital capacity, *HR* Hazard ratio, *IPF* Idiopathic pulmonary fibrosis

## Discussion

We have presented 95 AE-ILD patients who survived their first hospital treatment caused by AE-ILD. Furthermore, we have presented clinical data of 65 AE-ILD patients with at least 6 months’ survival after AE-ILD. We observed that a non-elective respiratory re-hospitalisation before the follow-up visit was an independent risk factor for mortality. Most patients still used corticosteroids at the six-month follow-up visit after AE-ILD. However, we were not able to observe a significant decline in PFT among AE-ILD survivors during the six-month follow-up period.

The overall mortality in AE-ILD is high, and approximately half of both IPF and other ILD patients die within three months after AE-ILD [25 − 26, 28, 31]. However, we observed that those who survived the acute hospital treatment caused by AE-ILD had a much longer median survival, namely 19 months, which suggests that some patients with AE-ILD have significant potential to recover.

Non-elective respiratory hospitalisation was a poor prognostic marker of AE-ILD survivors, which has not been reported in research settings similar to ours. According to Paternity et al. (2017), a respiratory-related hospitalisation was associated with an even higher risk for mortality than acute exacerbation among 1,132 placebo-treated study subjects in the nintedanib and pirfenidone programs [35]. However, our study material included AE-ILD patients only, and thus, is not comparable with the study mentioned above.

In our study, the usual cause of re-hospitalisation was clinical-radiological progression of AE-ILD, which also often resulted in death. Defining the exact cause of re-hospitalisation was challenging, especially differential diagnostics to acute infections versus natural disease course of AE-ILD, which share common symptoms and clinical findings. In the context of our study, re-hospitalisation could be regarded as an indicator of a more irreversible or aggressive phenotype of AE-ILD, often causing death.

We used information from death certificates to determine the causes of death. It should be noted that the practices for recording causes of death vary, and there is no specific ICD-10 code for AE-ILD. It is probable that AE-ILD was a major contributor to death in all 30 death cases observed during the 6 months’ follow-up, although the recorded cause of death was other than ILD in some individual cases.

It was reported by a Finnish study that patients with IPF spent 15% of their last 6 months of life in hospital and 80% of patients with IPF also died in hospital [36]. Our results might reflect the challenges in planning end-of-life care for patients with progressive ILD. The end-of-life decisions are often made late, and patients are treated in secondary or tertiary care even in the terminal phase of their disease, although, at least in Finland, end-of-life care should take place in primary care [36].

Hospitalisations of ILD patients are common, as has been reported in several earlier studies [37–41]. According to Pedraza-Serrano et al.(2019), 22% of hospitalised IPF patients experienced readmissions in 30 days after hospital discharge [38]. In this current study, half of the AE-ILD patients experienced re-hospitalisation in the 40 days after the hospital discharge, a proportion which is higher than in the study by Pedraza-Serrano et al., probably because our study population included AE-ILD patients only, not patients who had been hospitalised for any reason.

In our study, the majority of AE-ILD patients still used corticosteroids at the follow-up visit and continued the treatment afterwards at variable doses. This was also the case in the subgroup of 33 IPF patients, although current guidelines do not recommend corticosteroids or other anti-inflammatory drugs for IPF [42]. The optimal duration of corticosteroid therapy in the treatment of AE-ILD is not known. Farrand et al. (2020) reported that those patients who used corticosteroids during AE-IPF had increased mortality compared with those who did not use corticosteroids, which might even suggest that corticosteroids are not at all beneficial in AE-IPF [22]. In our study, all patients who died within six months after AE-ILD had used corticosteroids, whereas there were four patients who had been discharged without corticosteroids among the 6-month survivors. It is probable that those with more severe respiratory failure had been selected to be treated with corticosteroids, so one cannot draw any conclusions about the benefits of corticosteroids based on these results.

In contrast, Yamazaki et al.(2021) reported an association of an increased total dose of corticosteroids administered over one day to thirty days after AE-IPF with a decreased risk of recurrence of AE-IPF [43]. However, the corticosteroid dose after the first month of AE-IPF did no longer have an effect on the recurrence of AE-IPF [43]. Farrand et al. (2020) reported that the use of corticosteroid treatment did not influence 30-day readmissions among the 65 AE-IPF survivors [22]. In our study, only two AE-ILD patients were not treated with corticosteroids at any phase after the onset of AE-ILD, so the influence of corticosteroid treatment on re-hospitalisation cannot be evaluated. However, with regards to the median survival time of more than 1.5 years in this study, which is much longer than the typical overall survival in AE-ILD, it can be speculated that prolonged corticosteroid treatment has not been inevitably harmful for the AE-ILD survivors in our study.

There were only seven antifibrotic drug users among the AE-ILD survivors included in this study, although there were 90 patients with IPF who had received a reimbursement for antifibrotic drugs in OUH and OH areas by the end of 2017 according to the open database of the Social Insurance Institution of Finland (Kela) [44]. Pirfenidone received a recommendation for imbursement by Kela in 2013 and nintedanib in 2015. It should also be noted that during the implementation of this study, the reimbursements of antifibrotic drugs applied only to IPF patients, not to non-IPF patients with progressive pulmonary fibrosis. All patients of this study could not be offered antifibrotic drugs because they were not yet available during the first years of this study. It can also be speculated that those patients who used antifibrotic drugs experienced AE-IPF more rarely than those without antifibrotic treatment, which might further explain the small number of antifibrotic drug users in this study.

Decreased PFT results have been associated with a poor prognosis and increased risk for AE-ILD [23–26, 45–48]. Concerning this, it is surprising that the PFT results did not decline significantly among the study subjects on whom we had follow-up data after AE-ILD. As far as we are aware, similar PFT follow-up data related to AE-ILD has not been published before. Our results are encouraging for those who survive AE-ILD, indicating that the enhanced rate of decline in PFT is not an automatic consequence of AE. We observed a subgroup of ILD patients who had their diagnosis first time at the time of AE-ILD, and of whom 90% survived six months having better preserved PFTs compared with those of other study subjects. This suggests that AE-ILD occurred early in the disease in these patients and might explain their survival potential. Although AE-ILD is associated with poor prognosis in general, our findings suggest that patients with AE-ILD are a very heterogeneous group, and it is difficult to identify the individuals who have the capacity to recover from the episode of AE.

This study has several limitations. ICD-10 codes related to pulmonary fibrosis were utilised in the primary search of patients, so we may have missed some ILD cases whose treatment periods were not recorded with the ICD-10 codes that we used in the search. The study was retrospective in nature, which partially caused large amounts of missing data and made it challenging to compare the effectiveness of different drugs on AE-ILD in the absence of a control group. Furthermore, the effects of antifibrotic drugs on the course of AE-ILD could not be assessed due to the small number of antifibrotic drug users. Although the study design was retrospective, the collected data related to re-hospitalisations, follow-up visits, medical therapy, survival time and causes of death were comprehensive. Despite the limitations mentioned above, the clinical features of AE-ILD patients included in this study were similar compared with AE-ILD patients from other countries, which suggest that our results might be generalisable to international ILD patients as well [23, 30, 49].

## Conclusion

The outcome of the 95 AE-ILD survivors was variable because some of the patients had recovered and did not show progressed decline in their PFT while other patients died in less than six months, mainly because of ILD. Glucocorticoids were still used by 82.5% of patients at 6-month follow-up visit, although the usefulness of the treatment remained unclear. Respiratory re-hospitalisation was identified as a marker of poor prognosis that is easily recognised by the clinician and can guide clinical decision-making in the management of these seriously ill patients.

## Data Availability

The datasets generated and analysed during the current study are not publicly available due to the relatively small population of Northern Finland since we could not guarantee individuals’ anonymity as the data was collected in a detailed manner, but it is available from the corresponding author on reasonable request.

## Abbreviations

AE: Acute exacerbation
AE-ILD: Acute exacerbation of interstitial lung disease
AE-IPF: Acute exacerbation of idiopathic pulmonary fibrosis
BMI: Body mass index
CPAP: Continuous positive airway pressure
DLCO: Diffusion capacity for carbon monoxide
FEV1: Forced expiratory volume in the first second
FVC: Forced vital capacity
HFNO: High-flow nasal oxygen
HRCT: High-resolution computed tomography
ICD-10: International Classification of Diseases, Tenth Revision
ILD: Interstitial lung disease
IPF: Idiopathic pulmonary fibrosis
NIV: Non-invasive ventilation
NSIP: Non-specific interstitial pneumonia
OUH: Oulu University Hospital
OH: Oulaskangas Hospital
PFT: Pulmonary function test results
RA-ILD: Rheumatoid arthritis-associated interstitial lung disease
VC: Vital capacity
